# Skeletal Characterization of the *Fgfr3* Mouse Model of Achondroplasia Using Micro-CT and MRI Volumetric Imaging

**DOI:** 10.1038/s41598-017-18801-0

**Published:** 2018-01-11

**Authors:** Mohammed Salman Shazeeb, Megan K. Cox, Anurag Gupta, Wen Tang, Kuldeep Singh, Cynthia T. Pryce, Robert Fogle, Ying Mu, William D. Weber, Dinesh S. Bangari, Xiaoyou Ying, Yves Sabbagh

**Affiliations:** 1 Global Bioimaging Department, Translational In-vivo Models, Sanofi R&D Global Research Platform, 49 New York Avenue, Framingham, MA 01701 United States; 2 Rare Diseases, Sanofi, 49 New York Avenue, Framingham, MA 01701 USA; 3 Global Discovery Pathology, Translational In-vivo Models, Sanofi R&D Global Research Platform, 5 The Mountain Road, Framingham, MA 01701 USA; 4 Translational Sciences, Sanofi R&D Global Research Platform, 49 New York avenue, Framingham, MA 01701 United States

## Abstract

Achondroplasia, the most common form of dwarfism, affects more than a quarter million people worldwide and remains an unmet medical need. Achondroplasia is caused by mutations in the fibroblast growth factor receptor 3 (FGFR3) gene which results in over-activation of the receptor, interfering with normal skeletal development leading to disproportional short stature. Multiple mouse models have been generated to study achondroplasia. The characterization of these preclinical models has been primarily done with 2D measurements. In this study, we explored the transgenic model expressing mouse *Fgfr3* containing the achondroplasia mutation G380R under the Col2 promoter (Ach). Survival and growth rate of the Ach mice were reduced compared to wild-type (WT) littermates. Axial skeletal defects and abnormalities of the sternebrae and vertebrae were observed in the Ach mice. Further evaluation of the Ach mouse model was performed by developing 3D parameters from micro-computed tomography (micro-CT) and magnetic resonance imaging (MRI). The 3-week-old mice showed greater differences between the Ach and WT groups compared to the 6-week-old mice for all parameters. Deeper understanding of skeletal abnormalities of this model will help guide future studies for evaluating novel and effective therapeutic approaches for the treatment of achondroplasia.

## Introduction

Achondroplasia is the most common genetic form of dwarfism which affects more than 250,000 individuals worldwide^1,2^. Phenotypically, achondroplasia is characterized by a disproportionate short stature along with a large rounded skull. Significant complications of the disease include kyphosis, recurrent ear infections, and spinal fluid accumulation due to narrowing of the foramen magnum^3^. Low bone mass and bone density are also observed in adult and pediatric achondroplasia patients^4,5^. In addition, studies have shown that children with achondroplasia have mild cognitive delay^6^ as well as some structural changes to the brain^7^ that can be visualized by magnetic resonance imaging (MRI).

The genetic cause of achondroplasia has been found to be activating mutations in the Fibroblast Growth Factor Receptor 3 gene (*FGFR3*), the most common of which is G380R, which is found in more than 98% of achondroplasia cases^8^. The G380R mutation occurs in the transmembrane domain of FGFR3 resulting in stabilization of the receptor at the cell membrane leading to a ligand dependent increase in signaling^8–10^. FGFR3 has been shown to regulate chondrocyte proliferation and differentiation during bone growth^11–13^.

Multiple mouse models have been generated to investigate FGFR3 associated chondrodysplasias^12,14–16^. The two models that are often used for preclinical testing are: (1) the knock-in model of the thanatophoric dysplasia type I (TDI) mutation Y367C into the mouse *Fgfr3* gene, which displays an achondroplasia phenotype^14^, and (2) the transgenic model expressing mouse *Fgfr3* containing the achondroplasia mutation G380R under the type II collagen (Col2) promoter (Ach)^12^. Previous research into the Ach model has demonstrated that it exhibits the most prominent features of achondroplasia including rounded skulls and shortened limbs^12^.

Advances in imaging technologies, since the generation of the Ach mouse model, have provided new methods for visualizing and measuring skeletal abnormalities. The characterization of various preclinical models of achondroplasia has been primarily done with two-dimensional (2D) measurements using computed tomography (CT)^17–19^. Typical imaging parameters included linear bone lengths, linear craniofacial measurements of skull features^20–22^, and pedicle and lumbar vertebrae lengths^18^. Growth plate measurements were also commonly performed to quantify the growth plate heights using histomorphometry^23^. Trabecular bone analysis has also been done to quantify the microstructure of trabecular bone using parameters such as bone volume density, trabecular thickness, trabecular number, and trabecular separation^24–26^.

In this study, we developed and applied quantitative three-dimensional (3D) parameters from micro-CT and MRI to establish accurate and reliable imaging parameters for detailed characterization of the Ach model. These new measurements are important, especially the skeletal alterations in 3D, for accurate readouts of disease progression and treatment effects for potential new therapeutic modalities during the first 3 weeks of life in the Ach mouse.

## Results

### Fgfr3 expression

**Figure 1:**
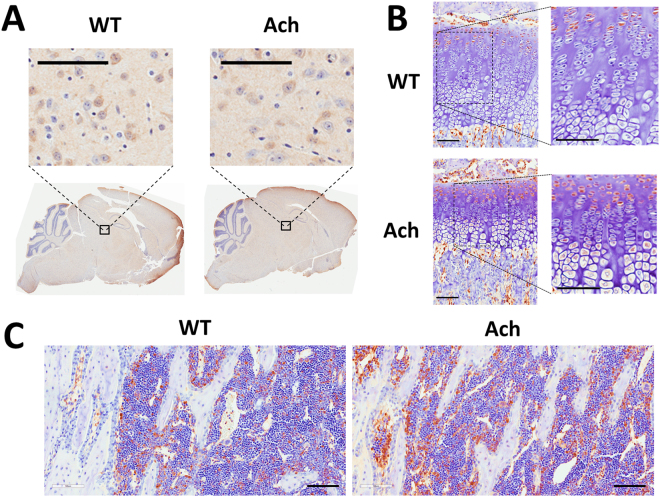
Fgfr3 expression in brain, cartilage, and bone of Ach mice. Immunohistochemical staining of Fgfr3 in the brain (**A**), growth plate region (**B**), and bone (**C**) of 3-week-old Ach and WT mice. Insets in (**A**) indicate thalamic nuclei. Insets in (**B**) show the growth plate region. Fgfr3 expression is shown by the brown stains in all the panels. Scale bars: 100 μm.

Immunohistochemical analysis demonstrated Fgfr3 expression in the brains of 3 week old WT and Ach mice (Fig. 1A) specifically in the multifocal neurons of the cerebral cortex, thalamus, and brain stem. No expression was seen in the cerebellum. As expected Fgfr3 expression was also seen in the proliferative chondrocytes (Fig. 1B) in both WT and Ach mice. The Ach mice had an increased expression of Fgfr3 which extended into the early hypertrophic chondrocytes (Fig. 1B). Fgfr3 expression was not detected in the bone cells but was seen in the bone marrow (Fig. 1C) of WT and Ach mice. Isotype controls are shown in Supplementary Fig. S1.

### Early onset of clinical signs and mortality in Ach mice

**Figure 2:**
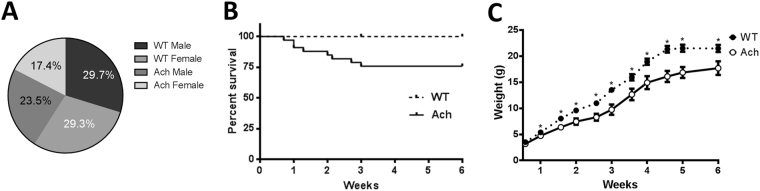
Survival and growth rates of WT and Ach mice. (**A**) Pie chart showing percentage of each genotype at one day after birth (n = 1560). (**B**) Survival plot showing attrition of Ach mice in first 6 weeks after birth [WT (n = 40), Ach (n = 33)]. (**C**) Body weight timeline of WT and Ach mice from birth to 6 weeks of age. Multiple t-tests using Holm-Sidak correction for multiple comparisons was used to show significant differences (*0.01 < *p* < 0.05) between the WT and Ach mice.

Ach males, which are heterozygous for the transgene, were crossed with wild-type (WT) FVB/NJ females. This cross would be expected to produce 50% WT and 50% Ach mice based on Mendelian genetics. The percentage of Ach mice actually born, deviated from these expected ratios (41% vs. 59%, chi-square *p* < 0.0001) and by day 1, approximately 25% of Ach pups died. This attrition seen at birth disproportionally affected female pups as seen in Fig. 2A (chi-square *p* < 0.0001). Mice that survived past day 1 still had increased mortality until weaning at 3 weeks of age (Fig. 2B) at which point any mice with paraparesis were euthanized. There was no additional mortality between 3 and 6 weeks of age. Significant reduction in bodyweight was seen by 1 week of age in Ach mice compared to WT mice and they continued to remain smaller through 6 weeks of age (Fig. 2C).

### Axial skeletal abnormalities in Ach mice

**Figure 3:**
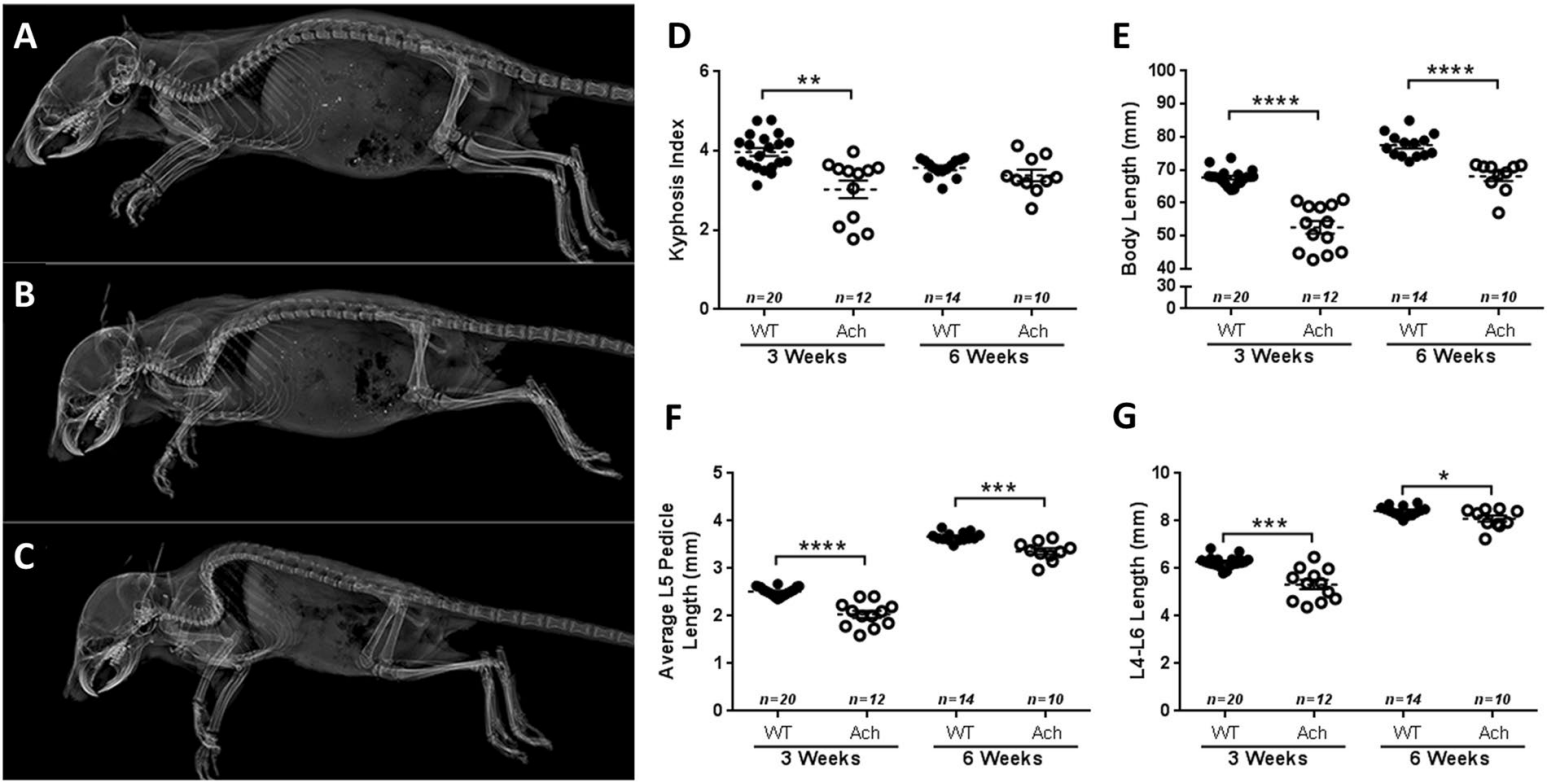
Axial skeletal defects in Ach mice. Lateral X-rays of 3-week-old WT (**A**) and Ach mice with mild (**B**) to severe (**C**) kyphosis. Kyphosis index (**D**) and body lengths (**E**) are shown for WT and Ach mice at 3 weeks and 6 weeks of age. Average L5 pedicle lengths (**F**) and L4-L6 lengths (**G**) are shown for the 3-week and 6-week-old mice. The graphical data are represented as mean ± SEM (*0.01 < *p* < 0.05, ***0.0001 < *p* < 0.001, ****p < 0.0001).

Ach mice showed varying degrees of kyphosis compared to WT mice (Fig. 3A) from mild (Fig. 3B) to severe (Fig. 3C) at 3 weeks of age. This increase in kyphosis is depicted by a significant decrease in the kyphosis index at 3 weeks of age (Fig. 3D, *p* = 0.0015). At 6 weeks of age, the WT and Ach mice did not show a significant difference in the kyphosis index. Most of the mice with severe kyphosis also displayed paraparesis which typically manifested around 2 weeks of age. The body lengths (Fig. 3E) of Ach mice were also significantly reduced compared to WT mice at both 3 weeks (*p* < 0.0001) and 6 weeks (*p* < 0.0001) of age, which indicate that this reduction did not necessarily occur due to kyphosis as the kyphosis index was not significantly different in 6-week-old mice. Along with the kyphosis and body lengths, Ach mice displayed significantly shortened lumbar (L5) pedicle length (Fig. 3F) and shortened lumbar vertebrae (L4-L6) length (Fig. 3G) as compared to WT mice at both 3 weeks (L5: *p* < 0.0001; L4-L6: *p* = 0.0004) and 6 weeks (L5: *p* = 0.0008; L4-L6: *p* = 0.04) of age.

**Figure 4:**
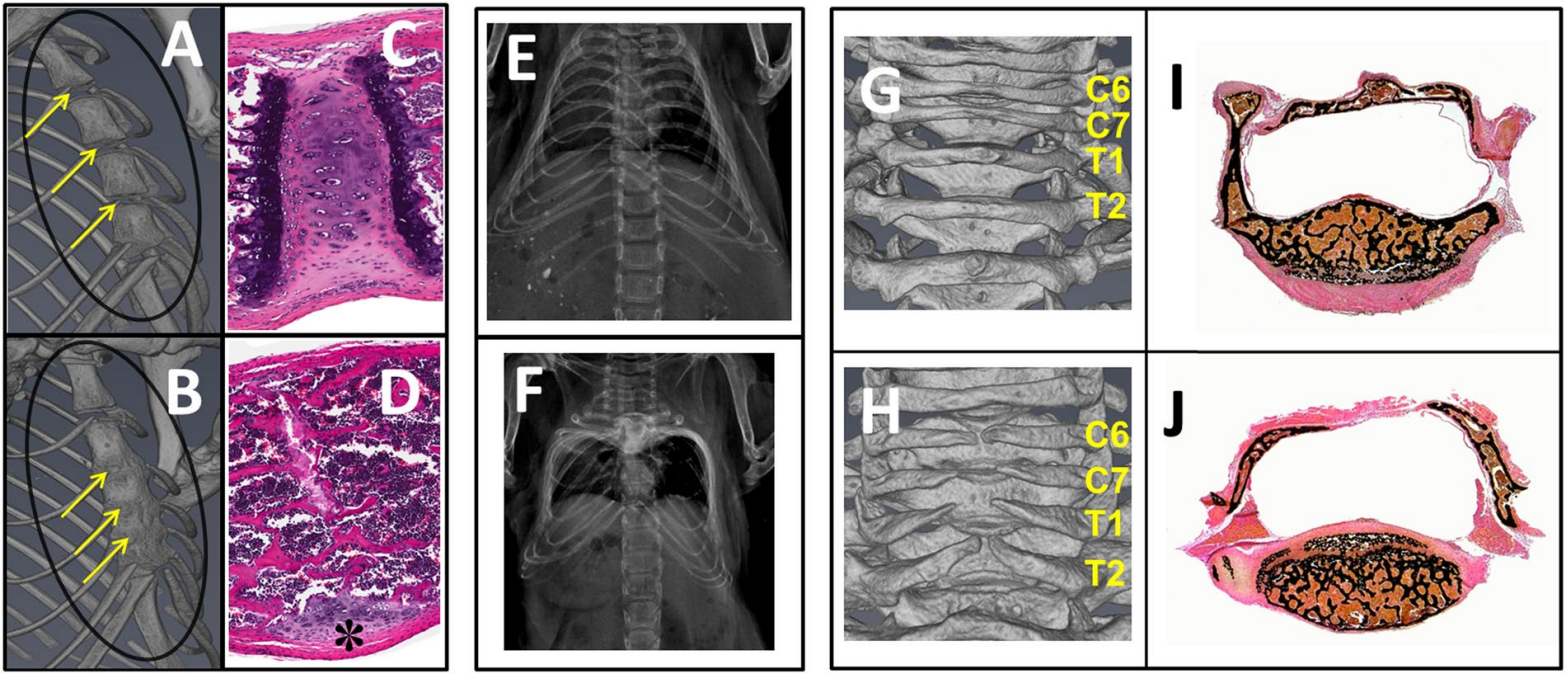
Abnormalities of sternebrae and vertebrae in Ach mice. 3D micro-CT of sternum with yellow arrows indicating junctions of adjacent sternebrae in representative 3-week-old WT (**A**) and Ach (**B**) mice. Corresponding H&E stained sections showing 3-week-old WT mouse (**C**) with joint cartilage and growth plate and Ach mouse (**D**) with mature bone replacing the intersternebral joint and growth plates. The asterisk shows receding joint cartilage. X-ray images are shown for the rib cages from representative WT (**E**) and Ach (**F**) mice at 3-weeks of age. Note lateral displacement of ribs giving rise to box-shape rib cage in Ach mouse. 3D micro-CT of vertebrae in representative 3-week-old WT (**G**) and Ach (**H**) mice reveal incompletely closed dorsal arch and lack of spinous process in Ach mouse. Corresponding von Kossa method stained sections show trabecular bone on the dorsal aspect of thoracic vertebra in WT mouse (**I**) and fibrous connective tissue instead of bone forming the dorsal arch in Ach mouse (**J**).

All Ach mice by 3 weeks of age had fusion of sternebrae, which was observed as early as 1 day post-natal (data not shown). Histologically, affected sternebrae revealed variable stages of joint and physeal fusion with complete atrophy of articular cartilage and replacement by modified trabecular bone. Early stage of fusion was characterized by fibrosis and necrosis whereas perpendicular alignment of trabeculae to endosteum and the receding remnant caps of costal cartilage were observed in the late stage of fusion in 3-week-old Ach mice (Fig. 4A–D), which continued to progress up to 6 weeks (Supplementary Fig. S2). The ribcage was malformed and appeared box-shaped, with lateral displacement of ribs and ventral deviation of sternum in severely affected Ach mice (Fig. 4E,F). Incomplete fusion of the dorsal arch of caudal cervical and cranial thoracic vertebrae and secondary lack of spinous process were observed in the Ach mice at 3 weeks of age (Fig. 4G,H). The Ach mice at 6 weeks of age showed a similar phenotype (Supplementary Fig. S2). Histology revealed fibrous connective tissue instead of bone within the dorsal arch region in Ach mice (Fig. 4I,J). Sternum and vertebrae of the WT mice were histologically unremarkable.

### Shortened tibia and femur lengths and decreased bone volume in Ach mice

**Figure 5:**
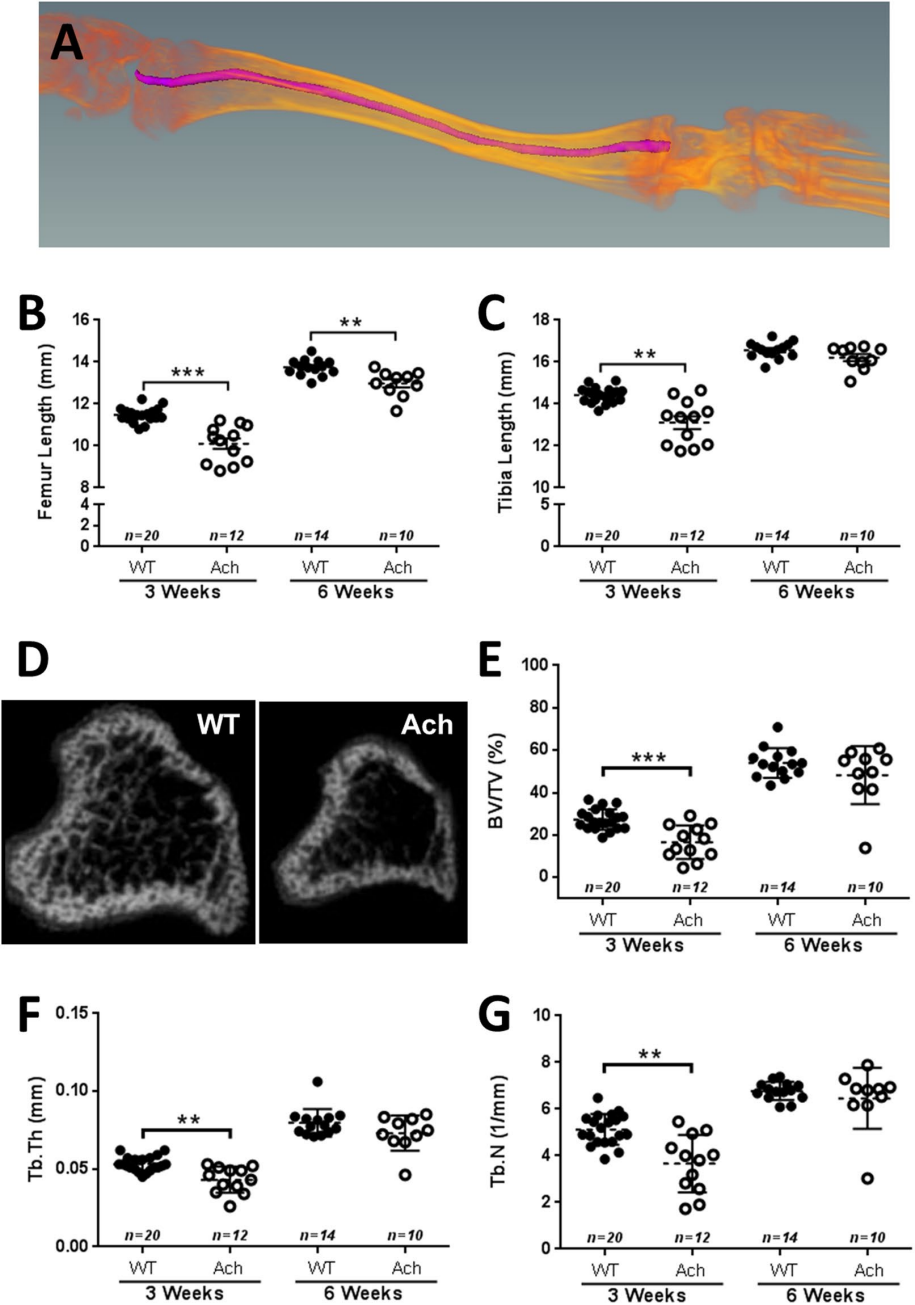
Micro-CT analysis of long bones in WT and Ach mice. (**A**) Illustration of curved tibia bone length measurements by micro-CT data. Summary of femur (**B**) and tibia (**C**) curved bone lengths are shown of 3- and 6-week-old mice. (**D**) Representative cross-sections of the tibia of WT and Ach mice are shown portraying the trabecular bone region. Summary of (**E**) bone volume density (BV/TV), (**F**) trabecular thickness (Tb.Th), and (**G**) trabecular number (Tb.N) are shown for 3- and 6-week old mice. The graphical data are represented as mean ± SEM (**0.001 < *p* < 0.01, ***0.0001 < *p* < 0.001).

Evaluation of femur and tibia lengths of WT and Ach mice at 3 and 6 weeks of age were done by 3D micro-CT (Fig. 5A–C). At 3 weeks of age, the Ach mice bone lengths were significantly shorter compared to WT mice for both the femur (*p* = 0.0002) and the tibia (*p* = 0.0011). At 6 weeks of age, the femur of Ach mice was significantly shorter compared to WT mice (*p* = 0.0038). In contrast, the tibia lengths between the WT and Ach mice were not significantly different at 6 weeks of age. Analysis of the trabecular bone region (Fig. 5D–G) showed a significant reduction in the bone volume density (BV/TV, *p* = 0.0006), trabecular thickness (Tb.Th, *p* = 0.0017), and trabecular number (Tb.N, *p* = 0.0017) in the 3-week-old Ach mice compared to WT mice. The 6-week-old mice did not show a significant difference in the trabecular analysis parameters between the Ach and WT mice (Fig. 5D–G).

### Growth plate abnormalities in tibiae of Ach mice

**Figure 6:**
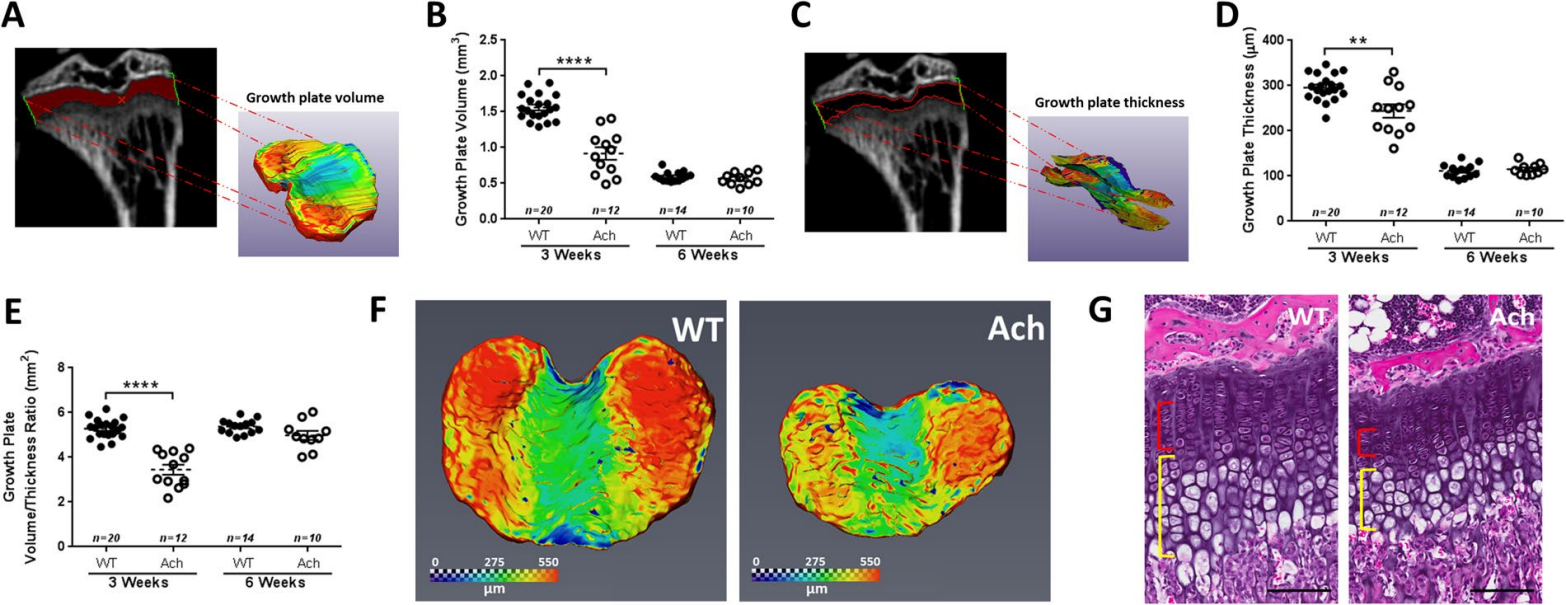
Micro-CT and histological analysis of the proximal tibia growth plate in WT and Ach mice. 3D reconstructions of the proximal tibia growth plates were generated from micro-CT images to calculate the growth plate volume (**A**,**B**) and thickness (**C**,**D**) in 3- and 6-week-old mice. The ratio of the growth plate volume to thickness is summarized in (**E**). (**F**) Representative growth plates from 3-week WT and Ach groups showing topographical contours of growth plate thickness. (**G**) H&E of 3-week-old growth plates show narrow proliferative (red bracket) and hypertrophic (yellow bracket) zones in Ach mouse compared to WT mouse. Scale bars: 100μm. The graphical data are represented as mean ± SEM (**0.001 < *p* < 0.01, ****p < 0.0001).

The proximal growth plates of tibiae in WT and Ach mice at 3 and 6 weeks of age were evaluated by micro-CT (Fig. 6). A significant decrease was observed in the Ach mice compared to the WT mice at 3 weeks of age for the following parameters: growth plate volume (Fig. 6A,B, *p* < 0.0001), growth plate thickness (Fig. 6C ,D, *p* = 0.0058) and the ratio of the two (Fig. 6E, *p* < 0.0001). The differences were not significant for any of the parameters at 6 weeks of age. The growth plate contours at 3 weeks of age (Fig. 6F) showed a thicker region along the lateral sides compared to the center of the growth plate for both WT and Ach mice. The H&E staining of the 3-week-old mice (Fig. 6G) showed narrow proliferative (red bracket) and hypertrophic (yellow bracket) zones in the Ach mice compared to the WT mice. This 2D-planar difference is in agreement with the 3D reconstructed measurements.

### Chondrocyte differentiation and proliferation in Ach mice

**Figure 7:**
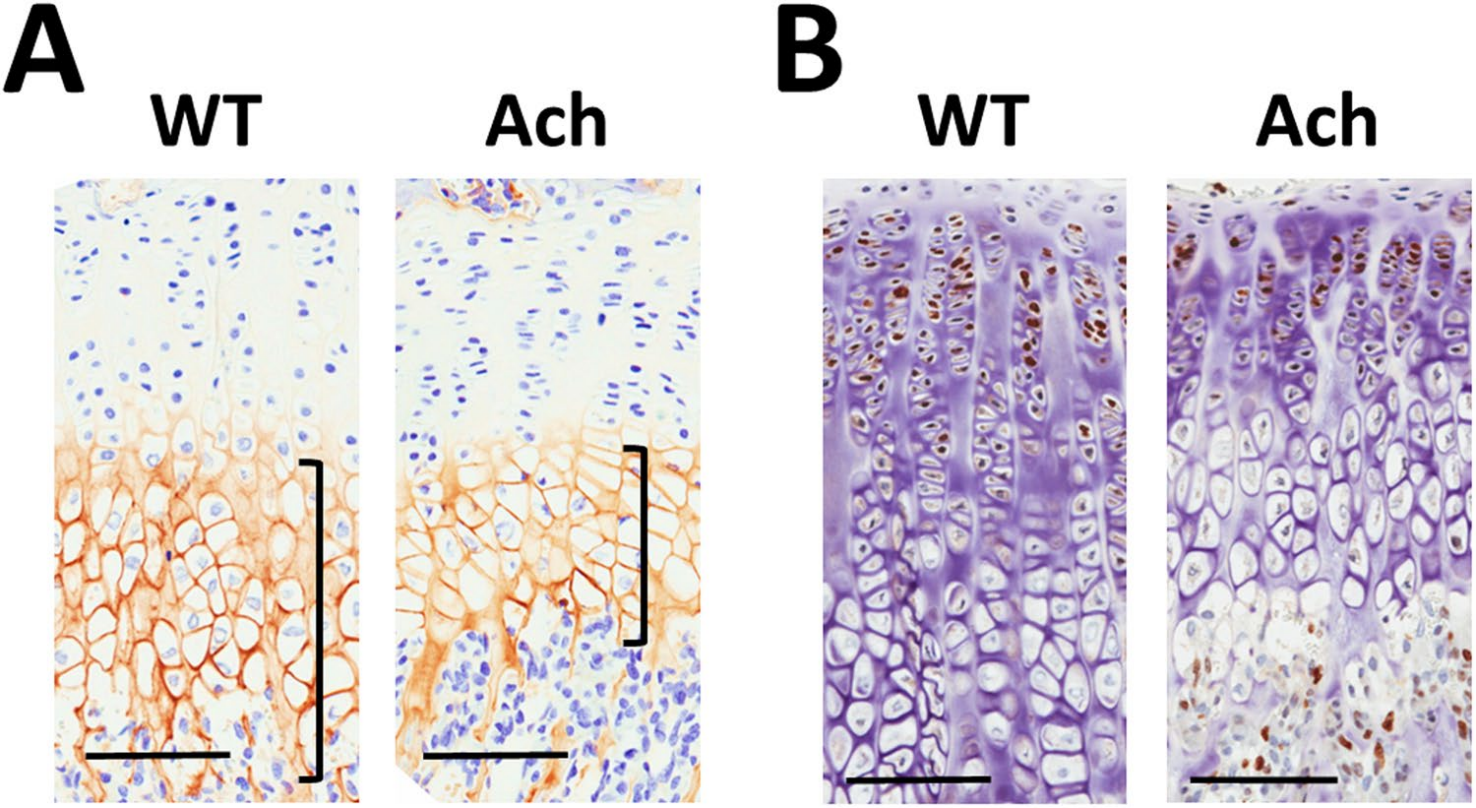
Chondrocyte differentiation and proliferation in WT and Ach mice. In the growth plate region, ColX (**A**) and Ki67 (**B**) expression are shown in 3-week-old Ach and WT mice. ColX protein expression is indicated in (**A**) by the brown staining in the hypertrophic region of the growth plate (brackets). Brown staining in the nucleus in (**B**) indicates Ki67 protein expression. Scale bars: 100 μm.

Decreased type X collagen (ColX) expression, a marker of differentiation, was seen in the hypertrophic region of the growth plate at 3 weeks of age for Ach mice compared to WT mice (Fig. 7A, Supplementary Fig. S3). By 6 weeks of age the ColX expression was no longer different between WT and Ach mice (Supplementary Fig. S3). Ki67 expression, a marker of proliferation, was clearly seen in the proliferative region of the growth plate for both WT and Ach mice at 3 weeks (Fig. 7B) and 6 weeks (Supplementary Fig. S3) of age. This region of Ki67 in the Ach mice was reduced when compared to WT mice at both ages. The difference between WT and Ach mice was less at 6 weeks of age compared to 3 weeks of age (Supplementary Fig. S3). Isotype controls are shown in Supplementary Fig. S1.

### Structural abnormalities in the brain and skull of Ach mice

**Figure 8:**
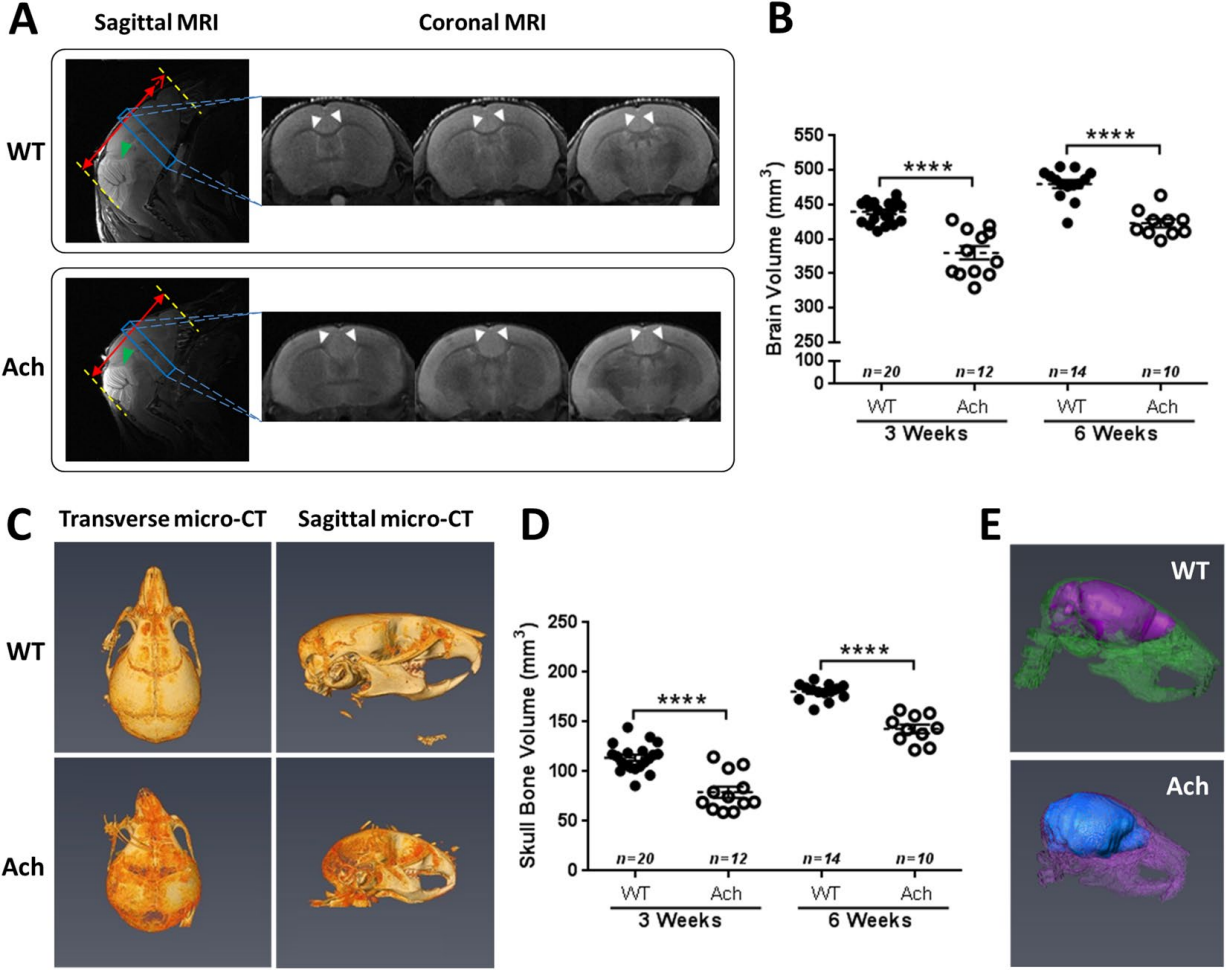
MRI and micro-CT analysis of brain and skull in WT and Ach mice. (**A**) Representative WT and Ach mice brains of 3-week-old mice are shown in the sagittal and coronal orientations. The red, green and white arrows indicate anatomical differences between the two groups. (**B**) Summary of the brain volumes using MRI are shown for 3- and 6-week-old mice. (**C**) Representative WT and Ach skull bones from 3-week-old mice are shown in the transverse and sagittal orientations. (**D**) Summary of the skull bone volumes using micro-CT are shown for 3- and 6-week-old mice. (**E**) A fusion of the brain MRI and skull micro-CT data are shown for better multimodal visualization. The graphical data are represented as mean ± SEM (****p < 0.0001).

Brains and skulls of WT and Ach mice were analyzed by MRI and micro-CT, respectively. On a sub-structural level, the cerebellum of the 3-week-old Ach mice appeared smaller than that of the WT mice (Fig. 8A, green arrows). The length of the brain in the sagittal orientation was shorter in the Ach mice compared to the WT mice (Fig. 8A, red solid lines are of equal lengths and the red dotted extension arrow in the WT mouse shows the extra length). The shape of the corpus callosum in the Ach mice was less prominent and had a diffuse edge (due to hypoplasia) compared to the WT mice (Fig. 8A, white arrows). The Ach mice also showed greater bowing of the corpus callosum in the center compared to the WT mice. MRI of the brains also showed a significant reduction in the brain volume of Ach mice compared to WT mice at both 3 weeks (*p* < 0.0001) and 6 weeks (*p* < 0.0001) of age (Fig. 8B).

The cranium in the Ach mice was relatively round compared to WT mice and some of the Ach mice exhibited a deviated nasal septum compared to that of the WT mice (Fig. 8C). The skulls of the Ach mice also showed a significant reduction in bone volume compared to the WT mice at both 3 weeks (*p* < 0.0001) and 6 weeks (*p* < 0.0001) of age (Fig. 8D). A composite image of the skull micro-CT and brain MRI data (Fig. 8E) appropriately captures the multimodal images for better visualization of the brain within the skull.

**Figure 9:**
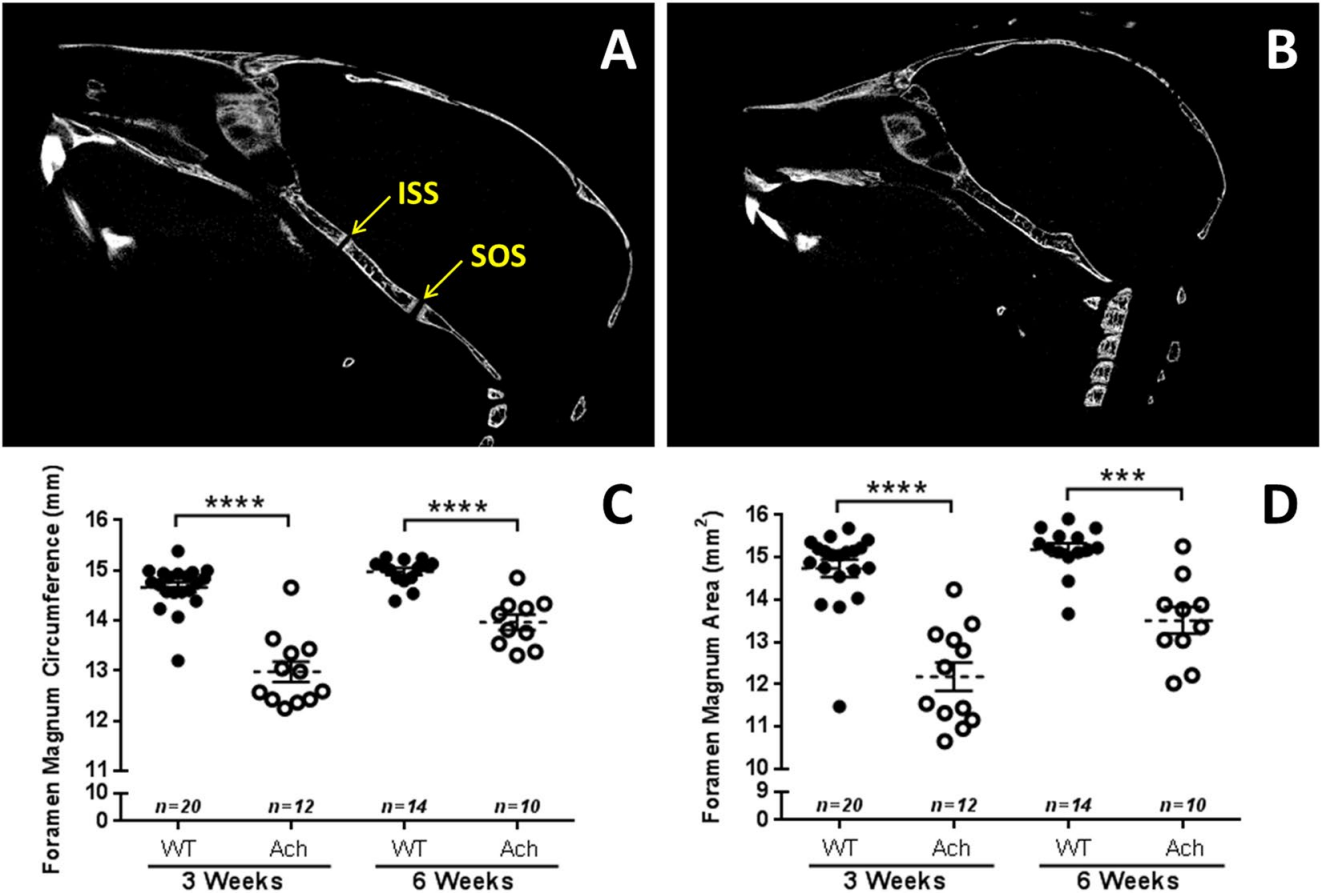
Micro-CT analysis of synchondroses and foramen magnum in WT and Ach mice. Representative WT (**A**) and Ach (**B**) mice skulls of 3-week-old mice show the fusion of the intersphenoidal synchondrosis (ISS) and the spheno-occipital synchondrosis (SOS) in Ach mice. Summary of the foramen magnum circumference (**C**) and area (**D**) from micro-CT measurements are shown from 3- and 6-week-old mice. The graphical data are represented as mean ± SEM (***0.0001 < *p* < 0.001, ****p < 0.0001).

The synchondroses (intersphenoidal synchondrosis and spheno-occipital synchondrosis) in the skull of Ach mice were prematurely fused compared to WT mice at 3 weeks (Fig. 9A,B) and 6 weeks (Supplementary Fig. S2) of age. The size of the foramen magnum was significantly reduced in the Ach mice compared to WT mice: the circumference of the foramen magnum (Fig. 9C) showed a significant reduction at both 3 weeks (*p* < 0.0001) and 6 weeks (*p* < 0.0001) of age. The area of the foramen magnum (Fig. 9D) also showed a significant reduction at both 3 weeks (*p* < 0.0001) and 6 weeks (*p* = 0.0004) of age in the Ach mice.

## Discussion

Despite advancements in understanding of achondroplasia in the past couple of decades, treatment options for patients that address the underlying problem are still not available. Current standard of care includes major surgeries to repair cervical cord compression and straightening bowed legs^1^. Pharmaceutical approaches using human growth hormones have also been used to increase patient height, though with limited efficacy^1^. Recent natural history studies have shown that patients have low bone mass^4,5^ and brain malformations with mild cognitive deficits^6,7^. There has also been progress investigating new therapeutics including various small molecules^18,27,28^, a soluble form of FGFR3^29^, and a modified C-type natriuretic peptide, which is currently in clinical trials^17,30^. Most of the aforementioned preclinical studies involved initiation of treatment within a few days after birth up to 3 weeks of age. This therapeutic window is important as it corresponds to the period of active and rapid growth and would translate to the patient population that would be amenable to treatment. Hence, our analysis focused on 3-week-old Ach mice. Further research into different non-invasive treatment modalities could lead to improvement in the quality of life for achondroplasia patients.

Since the original introduction of the Ach mouse model^12^, several advances have been made in achondroplasia research. Recently, Lee *et al*.^16^ published a new achondroplasia mouse model using a knock-in of the human *FGFR3* gene with the G380R mutation. They showed similar phenotypes (skull shape, body length, survival defects, bone density, and growth plate thickness) at 1 month of age compared to those observed in the Ach mouse model at 3 weeks of age. The Ach model showed increased Fgfr3 expression in the proliferative zone extending to the hypertrophic layer in the growth plate compared to WT mice, which is consistent with other achondroplasia models^12,14–16^. However, Fgfr3 expression in the brains showed little difference compared to the WT mice, which could be due to high Col2 expression in the brain occurring only in early development^31,32^. Therefore, the Fgfr3 detected at 3 weeks of age is possibly endogenous expression rather than the Col2-driven transgene.

In this study, we looked to develop new tools to significantly improve quantitative characterization of the Ach mouse model. While doing so, we encountered a high degree of phenotypic variability within the mice. We also explored the Ach mouse model in greater depth by performing a thorough characterization of different phenotypes with 3D imaging techniques using micro-CT and MRI. Other than mortality, no significant differences were observed between the sexes within each genotype. Therefore, the data were combined for all the other parameters analyzed.

The Ach mouse model has been previously described as having an increase in perinatal mortality^12^. We observed that this increased mortality begins from the day of birth and affects females more than males, which might have occurred due to the randomly inserted transgene causing a gender-specific effect. This increase in mortality has previously been attributed to respiratory failure^29^. Increased respiratory rate was observed in mice with malformed rib cages (Fig. 4F), suggesting that the skeletal deformity leads to labored breathing. Another potential cause of mortality was kyphosis (Fig. 3A–D), which was one of the more variable traits in the Ach mice. The most severely affected mice had the thoracic spine folding into the thoracic cavity and typically would progress to paraparesis leading to reduced mobility. During the first 3 weeks of age, Ach mice had significantly lower body weights than WT mice. When the Ach model was originally described by Naski *et al*.^12^, reduction in body weight was not seen until after 3 weeks of age. This earlier phenotypic observation could be the result of genetic drift resulting in more aggressive disease progression.

Quantification of vertebral measurements using a stereographic display in micro-CT provided easier localization of landmarks with greater consistency compared to performing skeletal preps or making 2D length measurements. Measurements like the L4-L6 length can be used to determine the effect of the achondroplasia mutation on the vertebra. Also, a reduction in vertebral pedicle length is seen in patients, clinically described as dystrophic pedicle syndrome, and can contribute to problems with compression of the spinal cord^33,34^. In the mouse model, these measurements are difficult to perform without high resolution tomography imaging due to the small size and tissue surrounding the vertebrae. Also, X-ray projections only allow 2D imaging of a 3D structure. Using micro-CT, we were able to detect a reduction in the pedicle length and a reduction in the lumbar vertebral length consistent with the clinical presentations in achondroplasia patients. We also observed a delay in the closure of the dorsal arch and lack of spinous process in the cervical and thoracic vertebrae. This delay in closure is similar to what has been shown in other mouse models with defects in proliferation^35^ or migration^36^ of sclerotome cells during early development of the spine. Col2 promoters, like the one used in the Ach mice, have been shown to turn on in sclerotome cells during development^37–39^. Fgfr3 has also been shown to be expressed in the same region in mouse development^40^. Since Fgfr3 is known to inhibit proliferation, it is probable that the delayed closure was due to the proliferation defects of sclerotome cells. The premature fusion of the sternum was clearly seen by micro-CT although difficult to detect on X-ray projection radiography images. This fusion may be due to dysregulated terminal differentiation of the hypertrophic chondrocytes resulting in earlier bone formation.

Shortened long bone length is one of the most prominent phenotypes of achondroplasia. In addition to shortened bones, achondroplasia patients exhibit an angular deformity which causes the legs to bow^41,42^. Typically, in preclinical models, straight bone lengths are measured as an endpoint. However, we chose to explore 3D curved bone length measurements to capture the bone bowing giving a more accurate measurement of the length deficiency in the disease state. The shorter curved bone lengths of the femur and tibia at 3 weeks of age follow the same pattern as the 2D straight bone lengths observed in previous studies where they are significantly shorter than WT mice^18,19,34^. Interestingly, at 6 weeks of age, a reduction in the curved bone length of the femur was observed in Ach mice compared to WT mice, but was not observed for the tibia curved bone length. Kyphosis index is another parameter that was significantly different at 3 weeks of age but not at 6 weeks. This lack of difference at 6 weeks of age could be due to the fact that mice displaying any signs of paraparesis were not kept past weaning at 3 weeks of age for humane reasons resulting in only larger mice with less severe disease progression being assessed at 6 weeks of age.

Bone mineral density (BMD) is another important parameter in assessing bone quality. It has been reported that achondroplasia pediatric and adult patients exhibit low BMD^4,5^. Previous results from other models of achondroplasia have shown a reduction in BMD, BV/TV, Tb.Th, and Tb.N values in the femur at 3 weeks of age^25^, and at 2 and 4 months of age^24^. A study in the Ach model, which we used in our analysis, also showed a reduction in the aforementioned trabecular parameters in the metaphysis of the proximal tibia at 18 weeks of age^26^. At 12 months of age, these differences were lost as the WT mice lost more bone^26^. Interestingly, these changes were seen using only 4 mice per group^26^ suggesting that the phenotypic variation may lessen with age possibly due to the increased mortality we saw in severely affected Ach mice. In our study, we saw a significant decrease in the BV/TV, Tb.Th, and Tb.N values of the metaphysis of the proximal tibia only at 3 weeks of age. By 6 weeks, we no longer saw differences between WT and Ach mice. These differences could reflect changes in age related bone loss^43^. It is possible that this normalization of bone parameters is due to a delay in bone age as is seen in other achondroplasia models^44^. However, by 3 weeks of age we saw no difference in bone age between WT and Ach mice (data not shown). It is possible that bone age is delayed in younger mice since Pannier *et al*. showed that even in the more severe *Fgfr3* ^*Y367C/*+^ model, bone age begins to catch up to WT by 3 weeks of age and fully normalizes by 6 weeks of age^44^.

The 3D growth plate measurement is a novel parameter introduced in this study. Current methods use 2D histomophometry to measure the growth plate thickness at a certain section of the growth plate. However, as viewed in Fig. 6C, the growth plate has a heterogeneous structure and can be accurately depicted by reconstructing the micro-CT data (Fig. 6A,C). The reduction of the growth plate volume and thickness in the 3-week-old mice but not in the 6-week-old mice is again likely due to reduced survival of the most severely affected mice. The ratio of the volume and thickness is a surrogate of the surface area which can capture the heterogeneous nature of the growth plate providing a more accurate depiction of the growth plate structure. The histology sections in Fig. 6G support previous data^12^ showing a reduction in the growth plate thickness. The histology sections also clearly outline the proliferative and the hypertrophic regions, which are delineated by Ki67 and ColX, respectively. Both these regions are reduced in the Ach mice at 3 weeks of age demonstrating that activation of Fgfr3 leads to inhibition of chondrocyte proliferation and differentiation in the growth plate. At 6 weeks of age only the proliferative region showed a reduction in the Ach mice. However, the size of the proliferative region was reduced from 3 weeks of age in Ach and WT mice as is also observed in the *Fgfr3* ^*Y367C/*+^ model^44^. One limitation of micro-CT is its inability to decipher these growth plate sub-regions.

The 3D volumetric measurements of the brain and skull showed the most significant changes between the WT and Ach mice in both 3 weeks and 6 weeks of age. Several studies have explored the use of linear craniofacial measurements to quantify changes in the skull^18,20,21,29^. In our study, we used the 3D volume of the skull bone as a comprehensive parameter for the disease which showed a greater change compared to linear measurements (data not shown). The synchondroses in the skull of Ach mice were prematurely fused similar to that observed in the sternebrae. In addition, the foramen magnum of Ach mice showed a significant reduction in size, which is of primary concern clinically in the achondroplasia population. Even though Lorget *et al*. used MRI in the knock-in model to look at the foramen magnum^17^, no other studies have explored the use of MRI to study the brain of Ach mice. Reduction of the brain size and changes in the morphology of sub-neuronal structures such as the corpus callosum might lead to cognitive and behavioral changes as reported in previous clinical studies^7,45,46^. The composite image of the brain (reconstructed from MRI data) and the skull (reconstructed from micro-CT data) in Fig. 8E indicates that the brain in Ach mouse is compressed within the skull preventing the brain from developing completely. The reduction of the brain size and other neuronal sub-structures like the corpus callosum and the cerebellum are likely secondary to the malformation of the skull and not an intrinsic effect of the brain since we did not see differences in Fgfr3 expression in the brain between WT and Ach mice. However, more research needs to be done to clarify the mechanism of the brain malformations observed in the Ach mice.

The Ach mouse model is a good preclinical model to study achondroplasia since it recapitulates the various clinical phenotypes up to 3 weeks of age, which can be visualized using 3D quantification of MRI/CT imaging parameters. The variability of body size and kyphosis severity makes this model similar to the human phenotype. However, this variability makes preclinical studies more complex requiring the use of larger number of animals in order to accurately detect changes to the phenotype. The early onset of paraparesis in a sub-population of these mice differs from what is seen in patients, although this may partially be due to early intervention to correct spinal alignment in children which would prevent the mobility loss. The loss of mobility in the hind limbs may also affect bone growth independent of the *Fgfr3* mutation. As long as these limitations are considered in the preclinical design, the comprehensive characterization of the Ach mouse model presented in this study using multimodal imaging can be used to quantify disease endpoints of achondroplasia and test the efficacy of potential treatment strategies more accurately.

## Methods

### Animal Model

All animal experiments were approved by the Institute Animal Care and Use Committee of Sanofi and were performed in accordance with relevant guidelines and regulations. Mice were maintained in a virus- and parasite-free barrier facility and exposed to 12-hour light/dark cycle. Mice were maintained on standard rodent chow diet (PicoLab Rodent Diet 20, #5053; LabDiet, St. Louis, MO, USA). All breeding was done using Ach heterozygous males crossed to WT FVB/NJ females^12^. Population analysis was performed on the entire colony for a total of 1560 mice. Mice were genotyped for the presence of the transgene on day of birth using a custom designed TaqMan assay (ThermoFisher Scientific, Part#4400294; forward primer: GTGCCTTCCCAACCATTCC; reverse primer: TGGGCGCGGAGCATAG; probe: TTATCCAGGCTTTTTGACAAC) to detect the presence of the segment of the human growth hormone gene, which is part of the transgene construct^12^. Fifty-six mice (male and female) were used in this study containing Ach and WT littermates at 3 and 6 weeks of age.

### X-ray Radiography

X-ray radiography imaging was performed immediately post-mortem using a Faxitron UltraFocus digital X-ray radiography system (Faxitron Bioptics, Tucson, AZ, USA). Dorsal and lateral images were taken at 1.5× and leg images were taken at 6×. All measurements were obtained using the Faxitron Bioptics imaging software.

### Micro-CT

Micro-CT imaging of the leg bones, vertebrae, and skulls were performed using a SkyScan 1176 scanner (Bruker, Kontich, Belgium). The mouse bodies and dissected long bones were fixed in 10% neutral buffered formalin and washed and stored in PBS in polypropylene syringes at 4 °C. Prior to scanning, samples were allowed to warm up to room temperature. The leg bones were scanned together in batches of 19 legs with settings of 50 kV, 500 μA, 8.6 μm pixel resolution, 0.3° rotation steps, and 4 frames average imaging with a 0.5-mm Al filter. The whole body with the skull and vertebrae were scanned in batches of 8, at 8.6 μm or 17.2 μm resolution with settings of 65 kV, 368 μA, 0.3° rotation steps, and 4 frames average imaging with a 0.5-mm Al filter. The acquired X-ray projections were reconstructed using the Skyscan NRecon software (V1.6.10.1) with beam hardening correction and post-alignment.

### Magnetic Resonance Imaging (MRI)

MRI of the brains was performed using a Bruker Biospec 70/30 horizontal bore system (Bruker, Billerica, MA, USA) equipped with 460 mT m^−1^ gradients and a 36-mm diameter volume radiofrequency coil (Animal Imaging Research, Holden, MA, USA). T2-images were acquired using RARE sequence (TR/TE = 5500/33 ms, 50 coronal slices, 0.5-mm thickness, matrix size = 256 × 256, FOV = 25 mm). A few animals were selected from the 3-week group and scanned using a 10-mm surface coil (Bruker, Billerica, MA, USA) to capture the brain details at a higher resolution.

### Histology

Tibiae and sternum were collected from 3-week-old mice in 10% neutral buffered formalin and decalcified in 20% EDTA for 2 weeks. Tissues were then routinely processed, embedded in paraffin, sectioned at 5 μm, and stained with hematoxylin and eosin (H&E). Non-decalcified sections of vertebrae from 3-week-old mice were fixed in 10% neutral buffer formalin, routinely processed, embedded in methyl methacrylate, sectioned at 5 μm, and stained with von Kossa method.

### Immunohistochemistry

Formalin fixed paraffin embedded mouse tibia sections, mouse brain sections, HEK293 cells, and HEK293 cells stably expressing mouse Fgfr3 were processed by automated immunohistochemistry staining using the BondRX autostainer (Leica Biosystems) following standard protocols. Briefly, the sections were baked for 30 minutes and deparaffinized with Dewax Solution (Leica, AR9222) before being subjected to an antigen retrieval process. For Mouse Anti-Collagen Type X (ColX) antibody (1:100, Quartett, 1-CO097-05), sections were enzyme retrieved with Proteinase K (Leica, AR9551) for 10 minutes at 37 °C followed by peroxide block (included in Bond Polymer Refine DAB Detection kit (Leica Biosystems, 9800)) and Rodent Block M (Biocare, RBM961H). Anti-ColX was diluted in prepared Antibody Diluent (Quartett, 400108191) and incubated for 30 minutes at room temperature. For Rabbit Anti-FGFR3 (1:10, Quartett, 1-FI049-07), sections were enzyme retrieved with 5 mg/mL Hyaluronidase (Sigma, H2126) for 10 minutes at 37 °C followed by peroxide block and Rodent Block M. Anti-FGFR3 was incubated for 60 minutes at room temperature. For Rabbit Anti-Ki67 (1 μg/mL, Cell Signaling, 12202), sections were antigen retrieved using Citrate (Leica Biosystems, AR9961) heated to 100 °C for 20 minutes followed by peroxide block. Anti-Ki67 was incubated for 15 minutes at room temperature. Mouse on Mouse HRP Polymer (Biocare, MM620H) (ColX) or Rabbit on Rodent HRP Polymer (Biocare, RMR622H) (Fgfr3, Ki67) were used as a secondary antibody. Bond Polymer Refine DAB Detection kit was used according to manufacturer’s instructions for all antibodies.

### Bone Length, Growth Plate Thickness, and Trabecular Bone Analysis

AMIRA (V6.0.1, FEI, Hillsboro, OR, USA) was used for all numerical analysis of bone lengths and growth plate thickness. The lengths of leg bones were measured using seed points along the bone and a 3D length tool in AMIRA as illustrated for a tibia in Fig. 5A. For growth plate measurements, the growth plate region was outlined and selected in the 2D growth plate slices, which were then rendered into a 3D volume (Fig. 6A) for volume calculations. The growth plate thickness was measured using a histogram tool after cropping the growth plate volume into two surfaces (Fig. 6C). The ratio of the growth plate volume and the growth plate thickness was also calculated which corresponds to the surface area of the growth plate.

CT-Analyzer (V1.13, Bruker, Kontich, Belgium) was used for the trabecular bone analysis. The image datasets were orientated in 3D to vertically align the longitudinal axis of each tibia. A region of interest (ROI) and a further volume of interest (VOI) were selected along the tibia axis for the trabecular bone based on the guidelines of the American Society of Bone and Mineral Research^47^. The top-end slice for the ROI was identified at 0.1 mm distally from the growth plate towards the diaphysis and the bottom-end slice was defined at 0.5 mm from the top-end slice. The ROIs were then automatically segmented by a macro-program using CT-Analyzer to form a VOI. CT image gray levels from 30 to 50 were tested, and the gray level of 40 was used for the trabecular bone segmentations in the CT image analysis. The bone volume density (BV/TV = bone volume of the sample volume/ total sample volume), trabecular thickness (Tb.Th), and trabecular number (Tb.N) were measured as the 3D volumetric outcomes for trabecular bone microarchitecture.

### Morphometric Analysis of the Brain, Skull, and Vertebrae

For MRI analysis, the brain slices were segmented in the coronal plane using Cheshire (V4.4.5, PAREXEL, Waltham, MA, USA) and then reconstructed to 3D volume using AMIRA to calculate the brain volumes. 3D morphometric measurements of the skull and vertebrae were performed using AMIRA. The volume of the bone encompassing the entire skull was measured by rendering the thresholded image of all 2D slices containing the skull bone into a 3D volume. A fusion of the brain MRI and skull micro-CT data was generated in AMIRA for better multimodal visualization (Fig. 8A).

To accurately assess the size and shape of the foramen magnum, the circumference length and area of the foramen magnum were measured in 3D since most of the structure does not lie on a flat 2D plane. The circumference length was measured along the surface geodesic path on the foramen boundary of the 3D rendered skull. The transverse and sagittal diameters (D_*t*_ and D_*s*_) of the foramen were also measured in 3D to calculate the area (¼ × π × D_*t*_ × D_*s*_) similar to a morphometric analysis described recently in a human study^48^.

Kyphosis index was calculated as previously described^49^. The body lengths were measured from the X-ray images starting at the tip of the nasal bone to the base of the first caudal vertebrae. To measure the mean L5 pedicle length, the 3D length tool was used to measure the length of the right and left pedicle from the apex of the pedicle to the base of the vertebral body. The curved 3D length of the L4-L6 vertebrae measurement was adapted from the curved 3D length measurement of the mouse long bones. The seed points were drawn in the vertebral foramen starting at the proximal end of the vertebral body in L4 and ending at the distal end of the vertebral body in L6.

### Statistics

Statistical comparisons were made using GraphPad Prism (V6.00 for Windows, GraphPad Software, La Jolla, CA, USA). Analysis of the sex and genotype variation was done using Chi-square test for goodness of fit. Two-tailed unpaired t-tests with Welch’s correction were used to check for significant differences between the WT and Ach groups at 3 and 6 weeks of age. Bodyweight significance was determined using multiple t-tests with Holm-Sidak correction for multiple comparisons. A probability of *p* < 0.05 was considered to be statistically significant (*0.01 < *p* < 0.05, **0.001 < *p* < 0.01, ***0.0001 < *p* < 0.001, ****p < 0.0001).

### Data Availability

The datasets that were generated and/or analyzed during the current study are available from the corresponding authors on reasonable request.

## Electronic supplementary material

Supplementary Material
