# Supplementary material for: Skeletal Characterization of the Fgfr3 Mouse Model of Achondroplasia Using Micro-CT and MRI Volumetric Imaging
Source: Sci Rep. 2018 Jan 11;8:469. doi: 10.1038/s41598-017-18801-0 (PMC5765052; doi:10.1038/s41598-017-18801-0)
Supplement: Supplementary file 1 — Supplementary Material [file 41598_2017_18801_MOESM1_ESM.pdf]

Supplementary Information

Title: Skeletal Characterization of the *Fgfr3* Mouse Model of Achondroplasia Using Micro-CT and MRI Volumetric Imaging

Authors:

[#]Mohammed Salman Shazeeb[1],
[#]Megan K. Cox[2],
Anurag Gupta[1],
Wen Tang[2],
Kuldeep Singh[3]
Cynthia T. Pryce[4]
Robert Fogle[1],
Ying Mu[1]
William D. Weber[4]
Dinesh S. Bangari[3],
*Xiaoyou Ying[1] (Co-corresponding author),
*Yves Sabbagh[2] (Co-corresponding author).

[1]Global Bioimaging Department,
Translational In-vivo Models,
Sanofi R&D Global Research Platform, 49 New York Avenue, Framingham, MA 01701, USA

[2]Rare Diseases,
Sanofi, 49 New York Avenue, Framingham, MA 01701, USA

[3]Global Discovery Pathology,
Translational In-vivo Models,
Sanofi R&D Global Research Platform, 5 Mountain Road, Framingham, MA 01701, USA

[4]Translational Sciences,
Sanofi R&D Global Research Platform, 49 New York Avenue, Framingham, MA 01701, USA

Corresponding address:

Xiaoyou Ying, Ph.D.,
Sanofi R&D Global Research Platform, 49 New York Avenue, Framingham, MA 01701, USA
E-mail: xiaoyou.ying@sanofi.com (3D imaging analysis queries)
Phone: 508-271-4730

Yves Sabbagh, Ph.D.,
Rare Diseases, Sanofi, 49 New York Avenue, Framingham, MA 01701, USA
E-mail: yves.sabbagh@sanofi.com (animal model queries)
Phone: 508-270-2505

[#]authors contributed equally
*co-corresponding authors

Supplementary Methods

Preparing Formalin Fixed Paraffin Embedded Cells for Immunohistochemistry (IHC).

Approximately 106 HEK293 cells and mouse Fgfr3 transfected HEK293 cells were collected separately, spun down, and rinsed with Phosphate Buffered Saline three times to rid of any media. Cells were fixed in Zinc-Formaldehyde for 10 minutes at room temperature. Again, cells were spun down and washed with PBS three times to remove media and placed in a 50˚C water bath. Cells were resuspended in 250µL of 2% agar solution and placed on ice for agar to solidify. The agar cell pellet was placed in a histology cassette and fixed in 10% neutral buffered formalin overnight before processing for paraffin embedding. IHC staining for ColX and Ki67 were quantified using Aperio ImageScope (Leica Biosystems).

Supplementary Figures

Supplementary Figure S1.

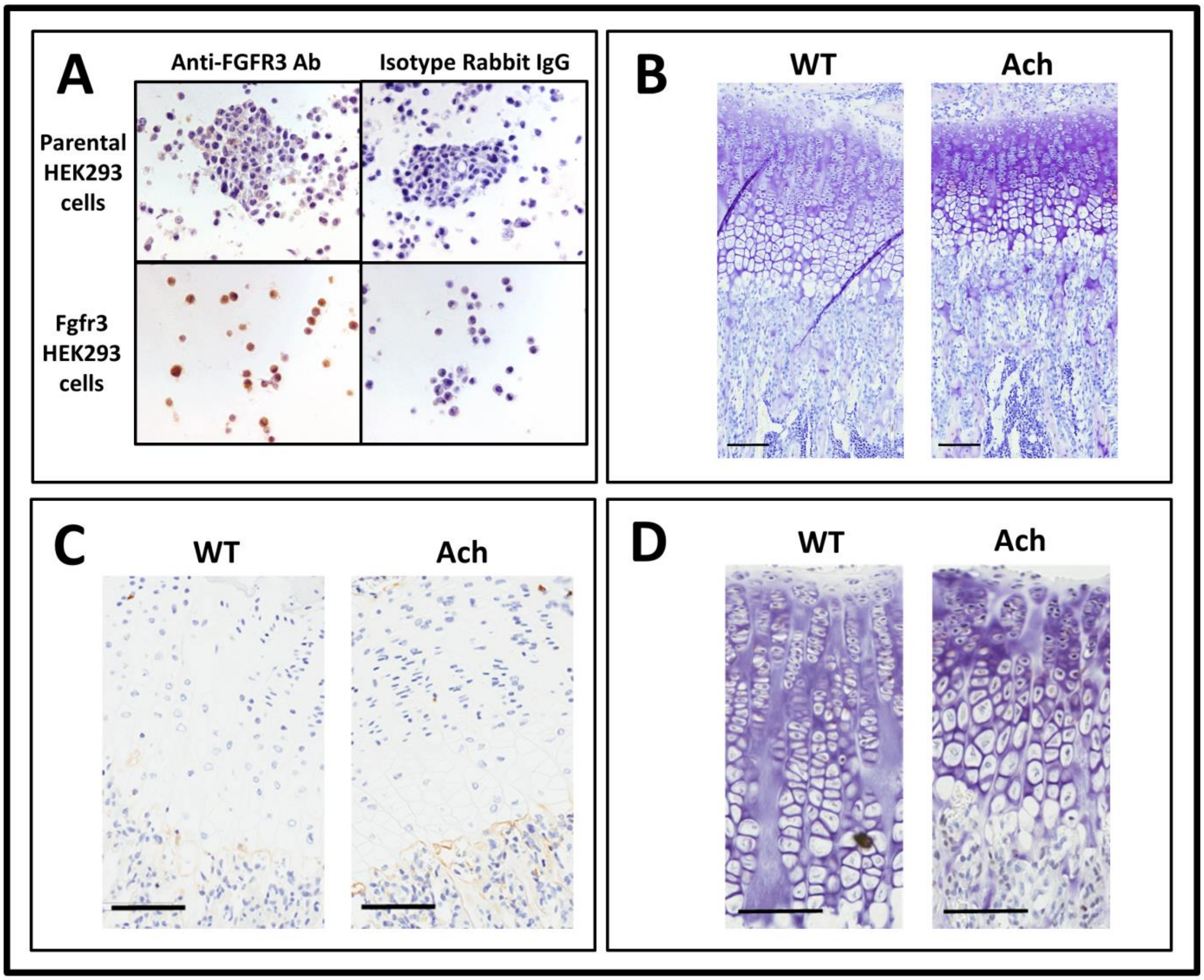

Supplementary Fig. S1. Controls for immunohistochemistry experiments. (**A**) First column shows immunohistochemistry staining for Fgfr3 in HEK293 cells. Parental cells show no specific staining (top) and cells stably expressing Fgfr3 show specific staining (bottom) indicating that the Fgfr3 antibody is specific for Fgfr3. Second column shows corresponding isotype rabbit IgG controls showing no nonspecific staining. (**B**) Isotype rabbit IgG control for Fgfr3 immunohistochemistry shows no nonspecific staining. (**C**) Isotype mouse IgG control for ColX immunohistochemistry shows minimal nonspecific staining. (**D**) Isotype rabbit IgG control for Ki67 immunohistochemistry shows no nonspecific staining. Scale bars: 100μm.

Supplementary Figure S2.

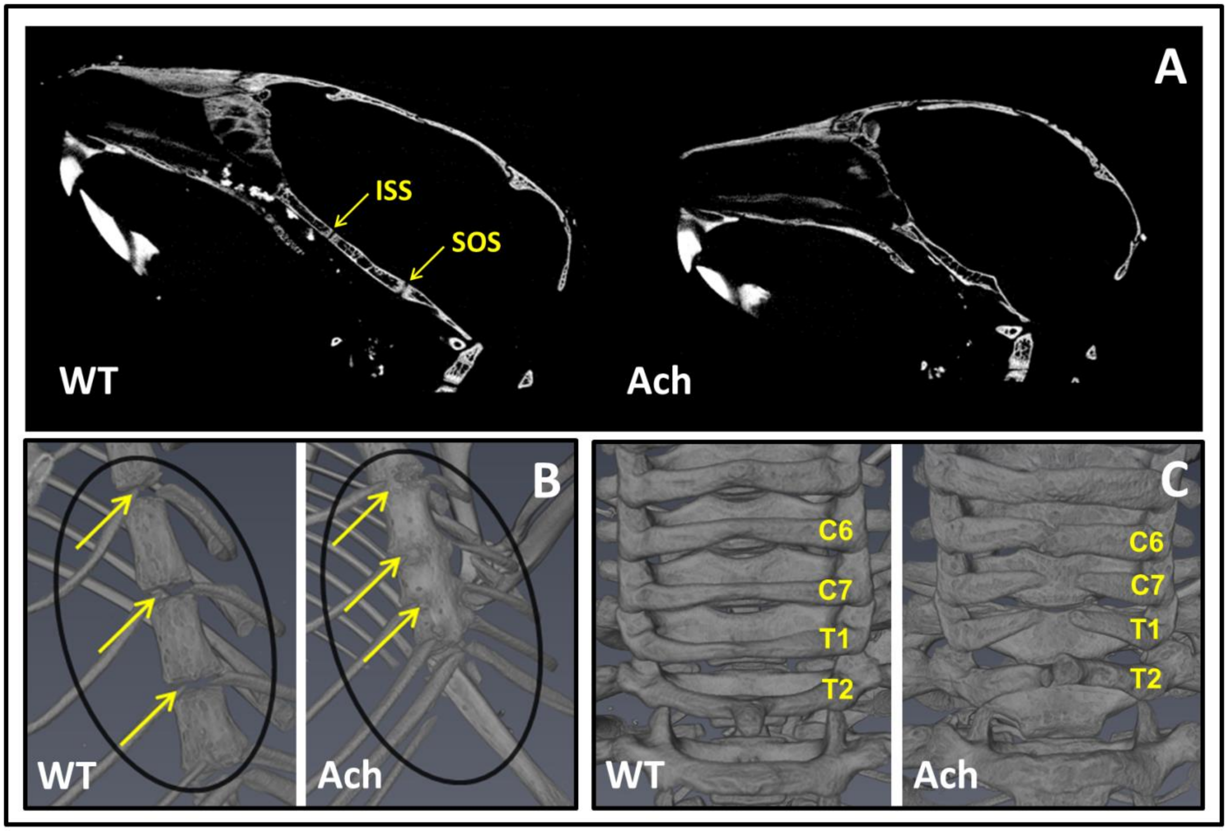

Supplementary Fig. S2. Abnormalities of synchondroses, sternebrae, and vertebrae in 6-week-old Ach mice. (**A**) Representative micro-CT images of WT and Ach mice skulls from 6-week-old mice show the fusion of the intersphenoidal synchondrosis (ISS) and the spheno-occipital synchondrosis (SOS) in Ach mice. (**B**) 3D micro-CT of sternum with yellow arrows indicating the junctions of adjacent sternebrae in representative 6-week-old WT and Ach mice. (**C**) 3D micro-CT of vertebrae in representative 6-week-old WT and Ach mice reveal incompletely closed dorsal arch and lack of spinous process in Ach mouse.

Supplementary Figure S3.

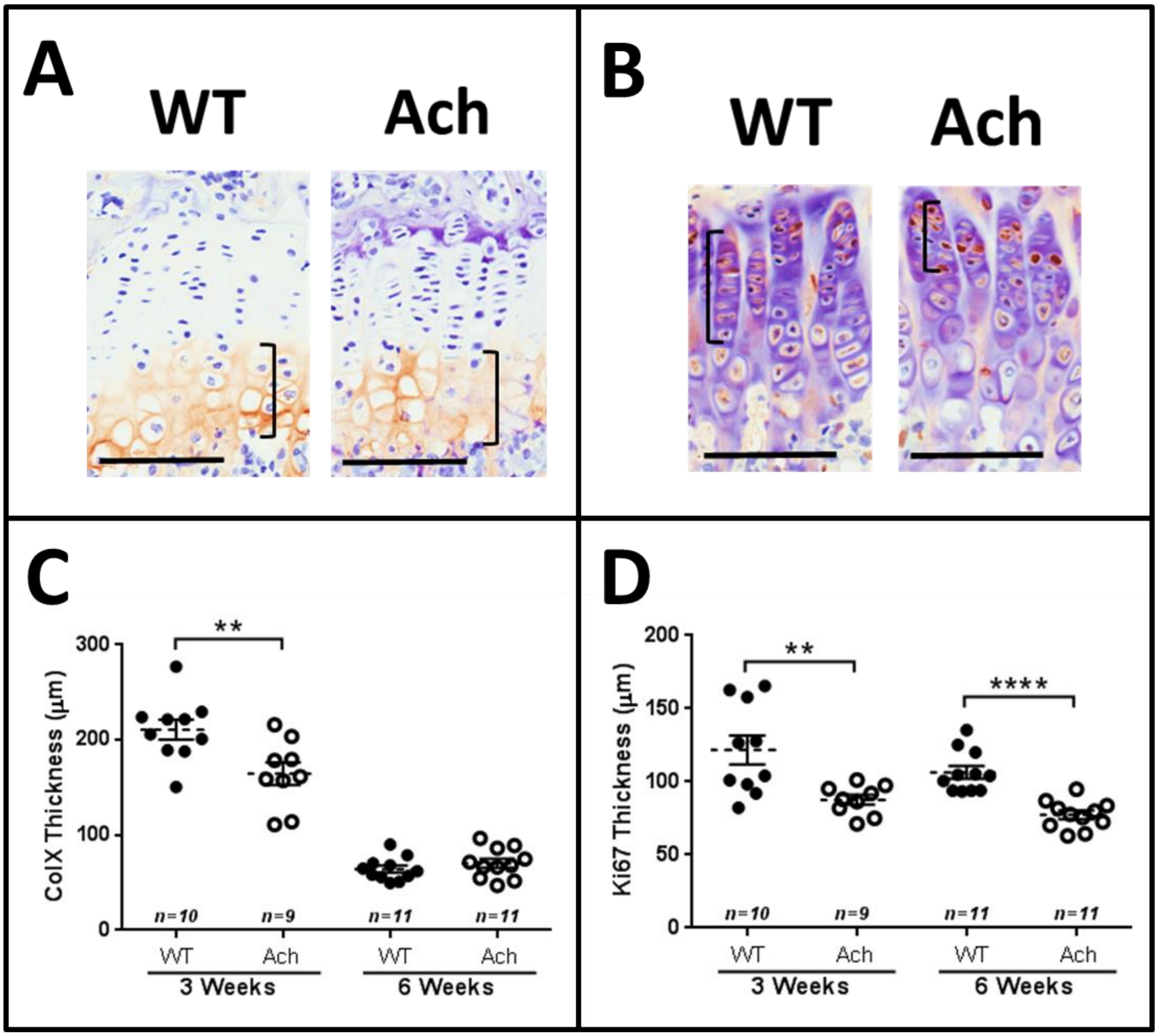

Supplementary Fig. S3. Histological quantification of chondrocyte differentiation and proliferation in WT and Ach mice. In the growth plate region, ColX (**A**) and Ki67 (**B**) expression are shown in 6-week-old Ach and WT mice. ColX protein expression is indicated in (**A**) by the brown staining in the hypertrophic region of the growth plate (brackets). Brown staining in the nucleus (brackets) (**B**) indicates Ki67 protein expression. Scale bars: 100µm. Quantification of the histological staining are summarized in (**C**) and (**D**) for ColX and Ki67, respectively, in 3-week and 6-week-old mice. The graphical data are represented as mean ± SEM (**$0.001 < p < 0.01$, ****$p < 0.0001$).
